# The Art of Rhinoplasty: Researching Technical and Cultural Foundations of Western World Rhinosurgery, from the Middle Ages to the Renaissance

**DOI:** 10.1007/s00266-021-02247-x

**Published:** 2021-04-19

**Authors:** S. Marinozzi, G. Sanese, D. Messineo, E. Raposio, L. Codolini, R. Carbonaro, V. Cervelli

**Affiliations:** 1 Department of Molecular Medicine, Unit of History of Medicine and Bioethics, Sapienza University of Rome, 34/a Viale dell’Università, 00161 Rome, Italy; 2 Department of Plastic and Reconstructive Surgery, Medical School, Tor Vergata University of Rome, Via Montpellier 1, 00133 Rome, Italy; 3 PhD School of Medical-Surgical Applied Sciences - Plastic Regenerative Research Area. School of Medicine and Surgery, Tor Vergata University of Rome, Via Montpellier 1, 00133 Rome, Italy; 4 Department of Radiological Sciences, Oncology and Anatomo-Pathological Science, Sapienza University of Rome, 31/33 Viale dell’Università, 00161 Rome, Italy; 5 Plastic Surgery Division, Department of Surgical Sciences and Integrated Diagnostics - DISC, University of Genova, L.go R. Benzi 10, 16132 Genova, Italy; 6 Unit of Plastic Surgery, Sapienza University of Rome, Viale del Policlinico 155, Rome, Italy; 7 International Medical School, Tor Vergata University of Rome, Via Montpellier 1, 00133 Rome, Italy; 8 Department of Plastic and Reconstructive Surgery, IRCCS Istituto Ortopedico Galeazzi, Via Riccardo Galeazzi, 4, 20161 Milan, Italy

**Keywords:** Rhinoplasty, Italian surgery, Italian rhinoplasty, Rhinosurgery, Vianeos, Tagliacozzi

## Abstract

The analysis of the written sources allowed to follow the gradual development of every new technique in the field of rhinoplasty but also to understand the value of this surgery in those ancient times, highlighting a deep connection between traumatologic surgery of the nose and the development of modern “aesthetic and reconstructive” Rhinosurgery. Specifically, we analyzed the techniques described by less known surgeons to emphasize their cultural and surgical value. As a matter of fact, the descriptions offered by these authors clearly show the importance of rhinoplasty as a cardinal and autonomous practice since Antiquity, also clarifying the persistence and development of specific techniques for this surgical practice in the History of medicine. In the manuscript, the contributions of the Italian surgeons, such as Brancas and Vianeos families, are highlighted, demonstrating their influence on the progress of this surgical specialty in the Early Modern Age. Finally, we deepen the description of Gaspare Tagliacozzi’s work, pointing out the topics and controversial debates arising from his techniques and innovations in “rhinosurgery” and also in the field of tissue transplantation, laying the foundations of modern Plastic Surgery.

*Level of Evidence V* This journal requires that authors assign a level of evidence to each article. For a full description of these Evidence-Based Medicine Ratings, please refer to Table of Contents or online Instructions to Authors www.springer.com/00266.

## Introduction

In the history of Western Medicine, the contribution and work of Arab doctors are fundamental. Heirs of ancient scientific knowledge, with the conquest of the Mediterranean basin, they bring back to the West the works of ancient Western authors, as well as the main treatises on practical medicine written by their most important medical experts. The Latin translation of these works allows a great diffusion not only of ancient theories, but also of the progress of surgical instruments and surgical techniques developed in the Islamic world. Avicenna (980–1037 A.D.), the epitome of Arab Medicine, still in the IX century proposes methods similar to those described by Hippocrates and Celsus. In case of fracture in the upper part of the nasal septum, a stylus (i.e., a stick for writing) is inserted in the nasal cavity, where the bone is refractured, to straighten it and then a packing, possibly of linen, is placed in the nostril. Unlike other authors, he prefers, however, not to bandage if there is no open wound. When, instead, the lower part of the septum is fractured, it is enough to insert the fingers to reopen the nostrils and reposition the bone. To treat any “*apostimas*” (i.e., edemas) and wounds, he uses the “*diachylon*” (i.e., a plaster made from medicinal herbs, lead oxide and rubber) or an ointment made from vinegar, oil, ash and burnt incense. In case of deformities due to fractures of the septum or the cartilages, one should bandage with a strip of linen soaked in fish glue and leather or rubber or other agglutinative, so that it adheres to the distorted part of the nose which should be pulled to the opposite side until it returns to its correct anatomical position; a bandage is then wrapped horizontally, tying the ends of it at the height of the nape of the neck [1].

The conquest of the Mediterranean basin by the Arabs leads, from a cultural point of view, to a great implementation in the scientific and naturalistic knowledge, especially in medicine. The ancient texts, which had been translated in Arabic in their integral version, are translated back to Latin,  and also the work of Arabic and Byzantine authors is translated into Latin.

It is possible that among these texts there was the translation of Sushruta Samhita [2]. The Arabic medicine was widely diffused from Asia to Africa and in Europe, especially in Spain, France and the south of Italy, where rhinoplasty found a renewed life, becoming a surgical discipline.

**Fig. 1:**
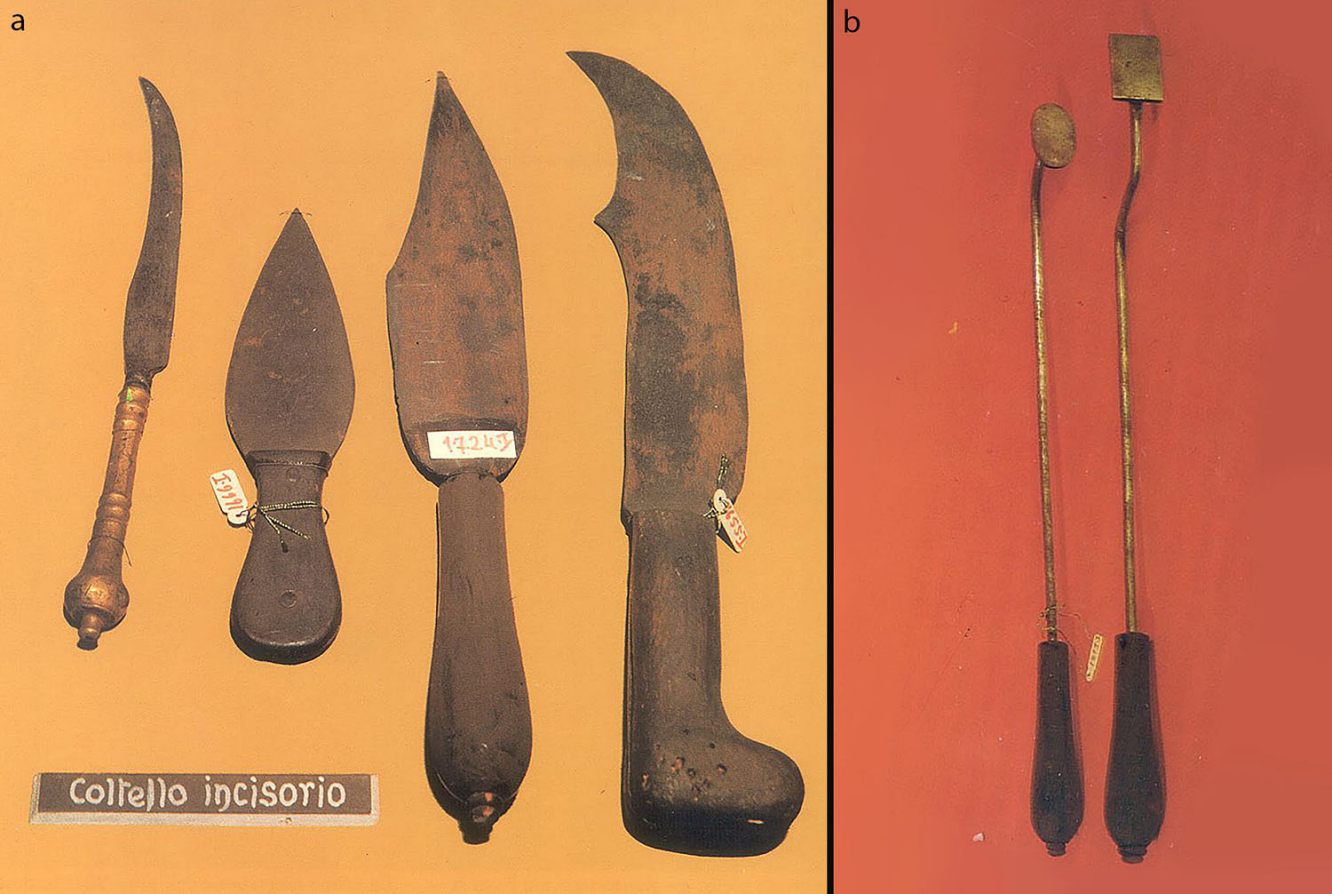
Medieval surgical instruments. **a** A scalpel and surgical knives used in the Middle Ages in Italy. **b** Cauteries used for hemostasis and welding during surgery (History of Medicine Museum—Sapienza University of Rome, Rome, Italy)

The liberalization and secularization of monastic schools in the late Middle Ages allowed the creation of medical schools, such as those of Montpellier in France and Salerno in Italy, which would give more vigor to practical medicine and surgery [3]. Surgery and surgical instruments in this period develop an increasingly relevant role within the curricula of the *Scholae Medicinae* (Fig. 1).

Hints on the restorative treatment of the nasal shape and function can be found in the work of Henry de Mondeville, surgeon of King Philip IV known as “the Handsome”, one of the leading exponents of the Medical School of Montpellier and famous for the development of new techniques and surgical instruments. In his *Surgery*, he describes the incisions that should be made in the region around the wound to cover it by means of traction of the two flaps of skin obtained as follows: *“Thus, when a small part of the nose is destroyed, incisions are made in the nearby region, so that they can come closer together using a strong pull, until they take root on the said loss of substance, cover it and, in a certain way, restore it.”* [4].

Particularly important for the improvement and the progress of rhinoplasty has been the contribution of Italian surgeons [5].

**Fig. 2:**
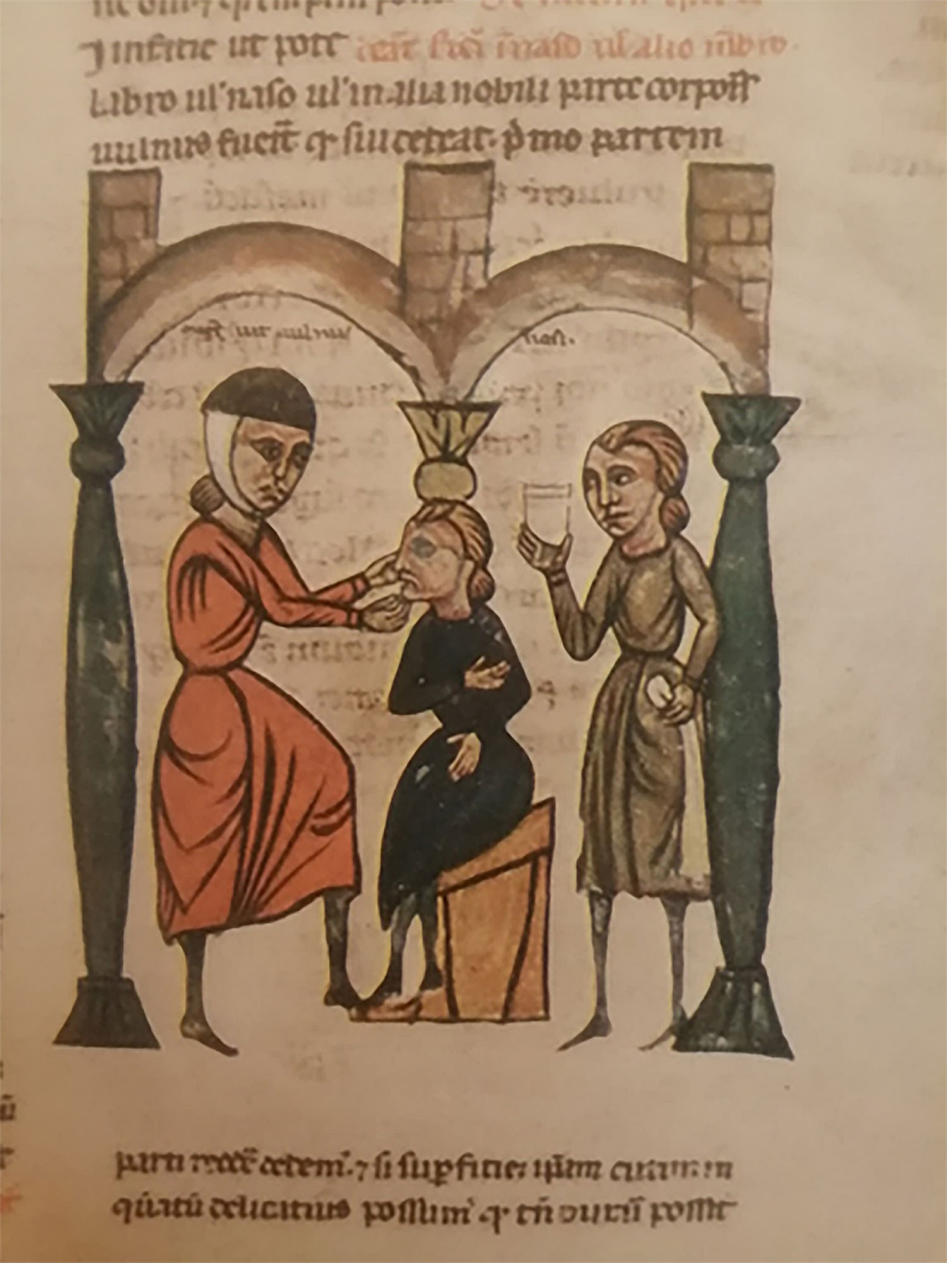
La Chirurgia di Rolando da Parma. Reproduction of Ms 1382 Casanatense Library of Rome. Rome: I.N.M.F., 1927, c.7v

In Italy Rolando dei Capezzuti, a member of the Medical School of Salerno, in his surgical work, mentions some precautions for the treatment and suturing of facial wounds, without however specifying the methods used in the practice of reconstructive surgery [6] (Fig. 2).

## Brancas and Vianeos: The First Rhinosurgery Specialists

Despite the interest shown and the descriptions provided by important exponents of official medicine, until the Fourteenth century rhinoplasty remains one of the many interventions described in treatises on general surgery, especially as a method of treatment for mutilations and wounds, without departing too much from the techniques of ancient authors. Lesser-known surgeons provided a precise doctrinal *corpus* that made plastic surgery of the nose a true surgical specialty, whose techniques were however jealously guarded and handed down from father to son in the families of those “practical surgeons” that “doctors” still consider as "laborers" compared to the courtly knowledge of official medicine.

Among the first Italians to devote themselves to rhinoplasty, there are the Brancas from Catania, whose fame was widely reported by historians of the time. The first information about the Brancas’ medical and surgical activity dates back to the early XV century, a period in which Gustavo Branca had already achieved considerable fame in the field of nasal reconstruction. There are not reliable proofs of the existence of other Brancas who devoted themselves to medicine and surgery prior to him and neither if there were family members experts in reconstructive surgery of the nose. According to certain historical-medical literature, they may have learned the art from some Arab surgeons. In contrast, according to some other historians, their method was innovative and was developed autonomously. Because of the similarity of their techniques with those of Indian surgeons, it was even suggested that there might have been a disclosure of Indian medical texts in Sicily, perhaps imported by Arab doctors. However, there is no real evidence to support one or the other hypothesis. It is probable that the criminal sanctions of nose amputation imposed by the constitution of the Kingdom of Sicily for the crime of adultery may have determined the development of a surgical technique for the reconstruction of the injured part.

While Gustavo Branca reconstructed the nose or a part of it by harvesting flaps of skin from the cheek, his son Antonio, in order to avoid disfigurements on the face, proceeded to harvest skin flaps from the medial-volar aspect of the arm to cover the lesions and to reconstruct the nostrils, using a sort of “two step flap” [6].

Even if rhinoplasty seems to have been abandoned with the death of Antonio Branca (1450), it reappeared in Calabria in the early 16th Century with the Vianeo family. Vincenzo is the first Vianeo, doctor or surgeon, of whom we have certain information concerning the practice of nasal plastic surgery. He then passed on the art to his nephew Bernardino. Certainly the most famous members of Vianeo family in this field are Paolo and Pietro, Bernardino's sons, who practiced their art in Tropea between 1540 and 1565 proposing the same technique used by Antonio Branca, consisting in harvesting of a strip of skin from the arm [7].

There are, however, no documents or historical evidence to support the hypothesis of the diffusion and transmission of the secret of Branca to other doctors, nor it is possible that Vincenzo Vianeo, who practiced the surgical profession between the end of ‘400 and the beginning of ‘500, was able to attend their interventions to learn the art [8].

It is certain that the work of these surgeons also aroused interest and admiration among some important members of official medicine, such as Alessandro Benedetti (1452–1512), an illustrious doctor and surgeon famous for his anatomic studies, who, in his most important work, also describes the technique used for the reconstruction of the nose:

*[...]These days some ingenious minds have shown how to correct the deformities of the nose: it has been seen several times that after cutting a piece of flesh from the patient's arm, they sewed it in the shape of nostrils and attached it to the mutilated nose. They separate the superficial skin from the arm with a razor, and after making the incision, they scrape, if necessary, the nostrils or they cut them again, then they tie the head to the arm so that the two wounds adhere to each other. When the wounds are “welded”, they finally cut from the arm, with a small knife, only what is necessary to reconstruct the nose: in fact, the small veins of the nose provide nourishment to the piece of flesh close to them. Finally, the skin is tailored over the nose […]* [9].

This paragraph describes simply and in just a few lines the important principle of autonomization of a flap prior to insetting, in a two-staged procedure known today as “delayed flap”. It is interesting to note that from this paragraph emerges a forerunner of the modern concept of flaps’ inosculation.

**Fig. 3:**
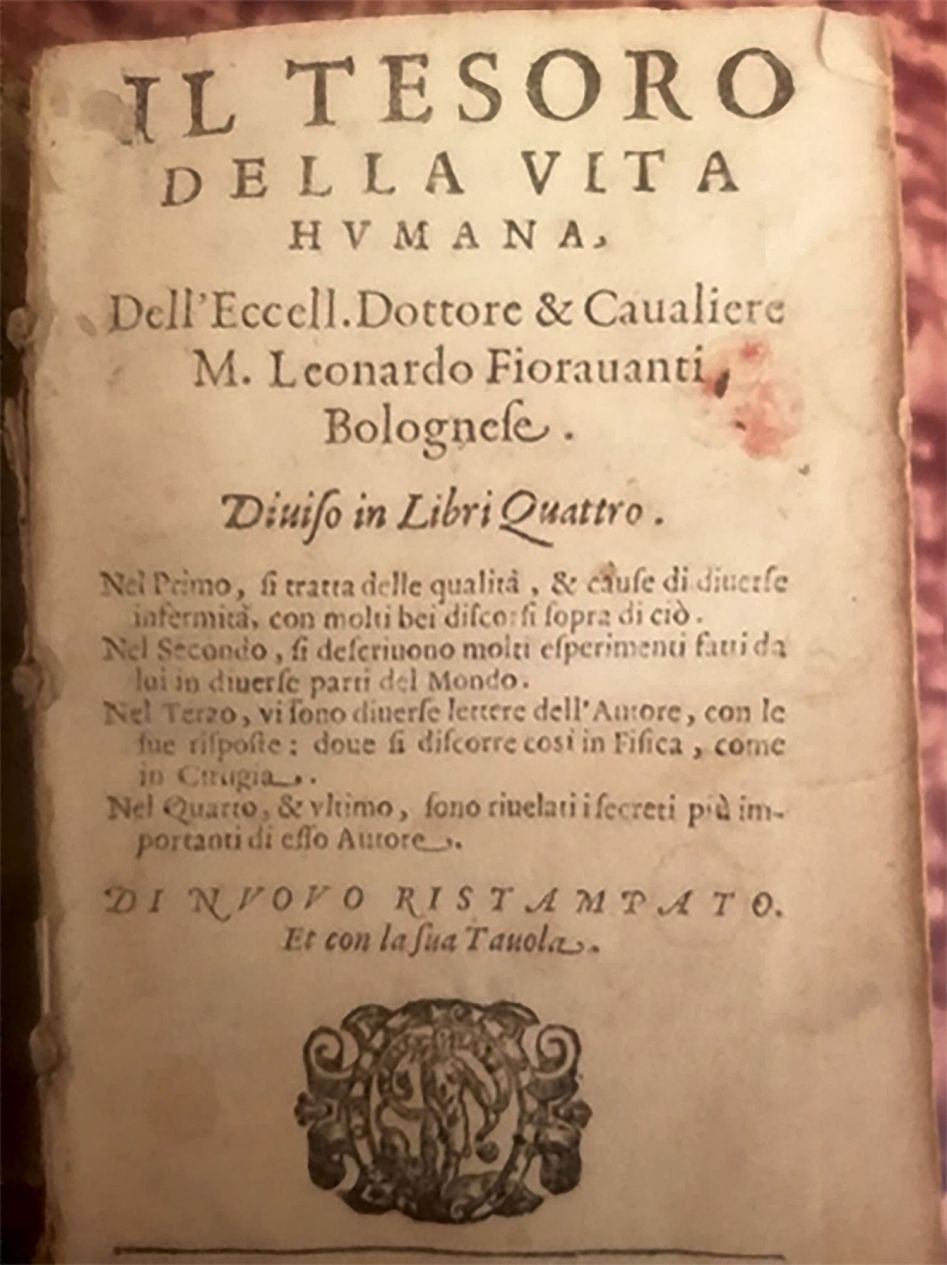
“Il tesoro della vita humana dell'eccell. dottore & caualiere M. Leonardo Fiorauanti bolognese”. In Venetia: for Spineda, 1629 (Biblioteca di Storia della Medicina, Sapienza University of Rome, Rome, Italy)

An exhaustive description of the technique used by the Vianeos is provided by Leonardo Fioravanti, who traveled both in Southern Italy and in Africa, staying also in Calabria, and specifically in Tropea, where the Vianeos practiced. In fact, he reports that he witnessed an operation carried out publicly by the two doctors (Fig. 3):

*[...]la prima cosa che costoro facevano ad uno quando li volevano fare tale operazione, lo facevano purgare, et poi nel braccio sinistro, tra la spalla e il gomito, nel mezzo pigliavano questa pelle con una tanaglia, et con una lancetta grande passavano tra la tanaglia et la carne del muscolo (…), et la medicavano sin tanto che questa pelle diventava grossissima, et come pareva a loro che fosse grossa a bastanza, tagliavano il naso tutto pare, et tragliavano questa pelle ad una banda et la cusivano al naso et la ligavano con tanto artificio et destrezza che non si poteva movere in modo alcuno sin tanto che la detta pelle non era saldata insieme col naso…[...]*

*[...]The first thing they did to one when they wanted to do this operation, they had him purge, and then in the left arm, between the shoulder and the elbow, in the middle they took the skin with a pincer, and with a large blade they passed between the pincer and the flash of the muscle (…), and they medicated it so that this skin became very plump, and as it seemed to them that it was big enough, they cut the whole nose, it would appear, and they cut this skin into a band and they sewed it to the nose and they tied it with so much artifice and dexterity that it could not move in any way until the said skin was not welded together with the nose…[...]* [10].

In the same work he reports that he witnessed a fight, in Africa, during which a gentleman lost his nose and that, once washed it and stitched it back, it was perfectly reattached [11].

The name Fioravanti is linked to the literary tradition of the *"Libri dei Segreti" (Books of Secrets)*: texts rich in notions of practical medicine and therapeutics, precepts of cosmetics and prevention, pharmacological and culinary recipes. These texts developed especially with the spread of alchemical art, which has a significant role in Spagyric medicine. They are written in the vernacular language so that they could also be accessible to non-academic professional categories, such as apothecaries, surgeons, empirical and practical doctors, and even to an elected but not expert public. They represented a tool for the dissemination of medical practices and treatment modalities for daily and widespread problems (such as acne, impetigo, wounds, ulcers, pediculosis, or mild disorders of the digestive, urinary or respiratory tract). These texts also contain hygiene and prevention precepts to indicate a healthy and correct lifestyle regimen that promotes health.

The widespread diffusion of these texts may suggest that the real Books of Secrets, like those of Leonardo Fioravanti himself, may have marked the passage of rhinoplasty from the empirical practice of Branca, Vianeo and other families of surgeons to the academic medicine of Gaspare Tagliacozzi (1546–1599 A.D.).

## The Academic Approach to Rhinoplasty: Tagliacozzi

Still a simple "handmaiden" of Medicine, with the affirmation of anatomy, in the midst of the Renaissance, surgery began to become an essential discipline in the training of doctors, with its own technical and doctrinal corpus, opening the way to surgical specialties.

 This allowed Gaspare Tagliacozzi (1547–1599 A.D.) to give a scientific basis to rhinoplasty in his "De curtorum chirurgia per insitionem". In his work, he proposes, in academic courtly language, techniques, methods and therapeutic systems that had already been adopted for some time in the practice by lesser-known surgeons such as the Brancas and Vianeos, thus laying the foundations for plastic surgery of the nose in a modern sense [12]. Pupil of Julius Caesar Aranzi, an anatomist and surgeon who devotes an entire part of his work to the surgical treatment of facial injuries, Tagliacozzi has perhaps drawn on the experience acquired by Fioravanti in his travel to Tropea, once he returned to Bologna [13]. He accurately describes the surgical procedure he used in rhinoplasty: incisions were made in the medial-volar side of the arm to rise a flap of skin that was then positioned over the part of the nose that has to be reconstructed. The flap was not harvested completely, but it was left to survive on an inferiorly based vascular pedicle.

**Fig. 4:**
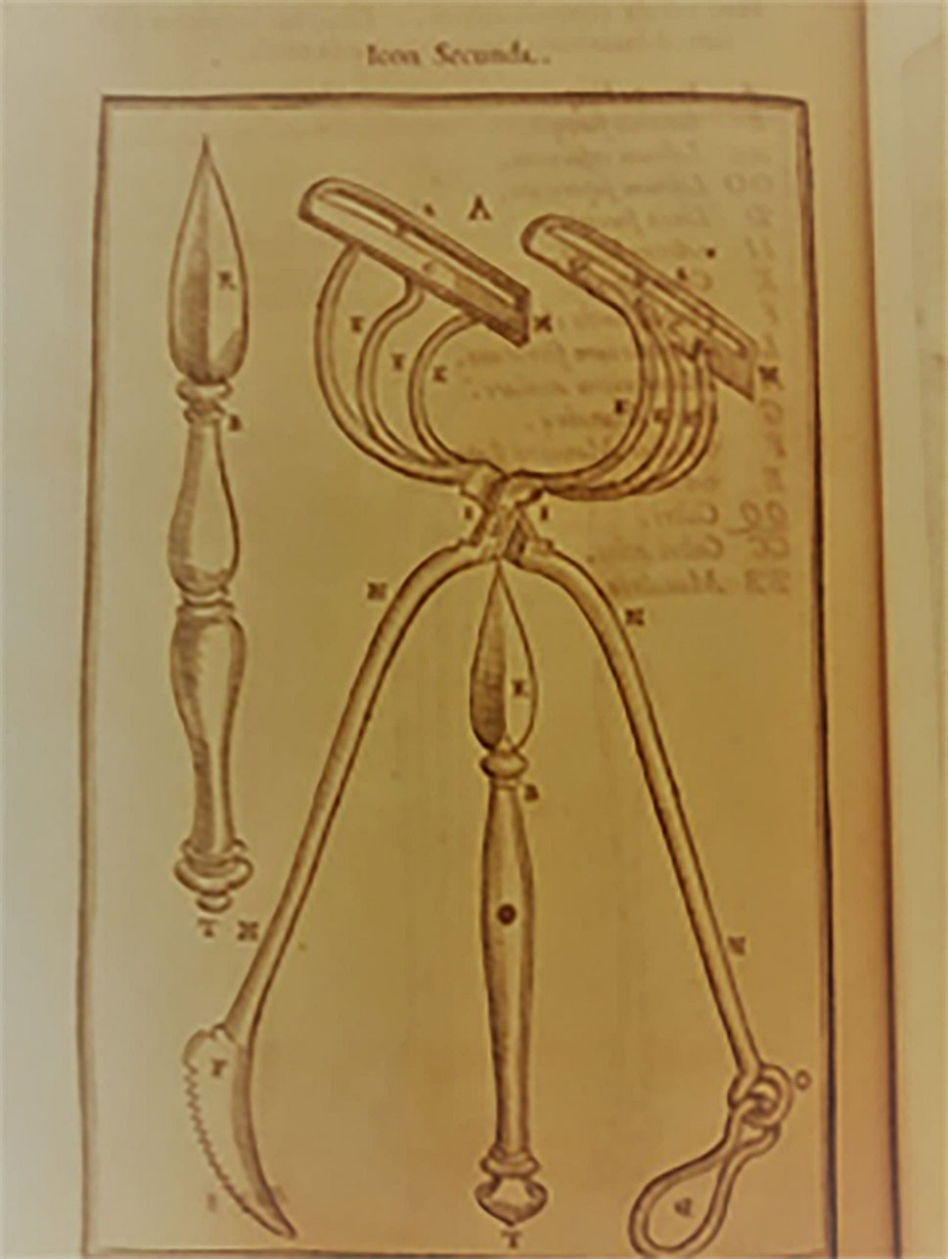
Surgical instruments, tissue forceps used to hanlle the cutaneous flaps during Tagliacozzi’s procedure; Table 1, *De Curtorum chirurgia per insitionem, additis instrumentorum omnium et delegationum iconibus, et tebulis libri duo”.* Venezia, Bindoni, 1597, Tables 1 (Library of History of Medicine—Sapienza University of Rome)

**Fig. 5:**
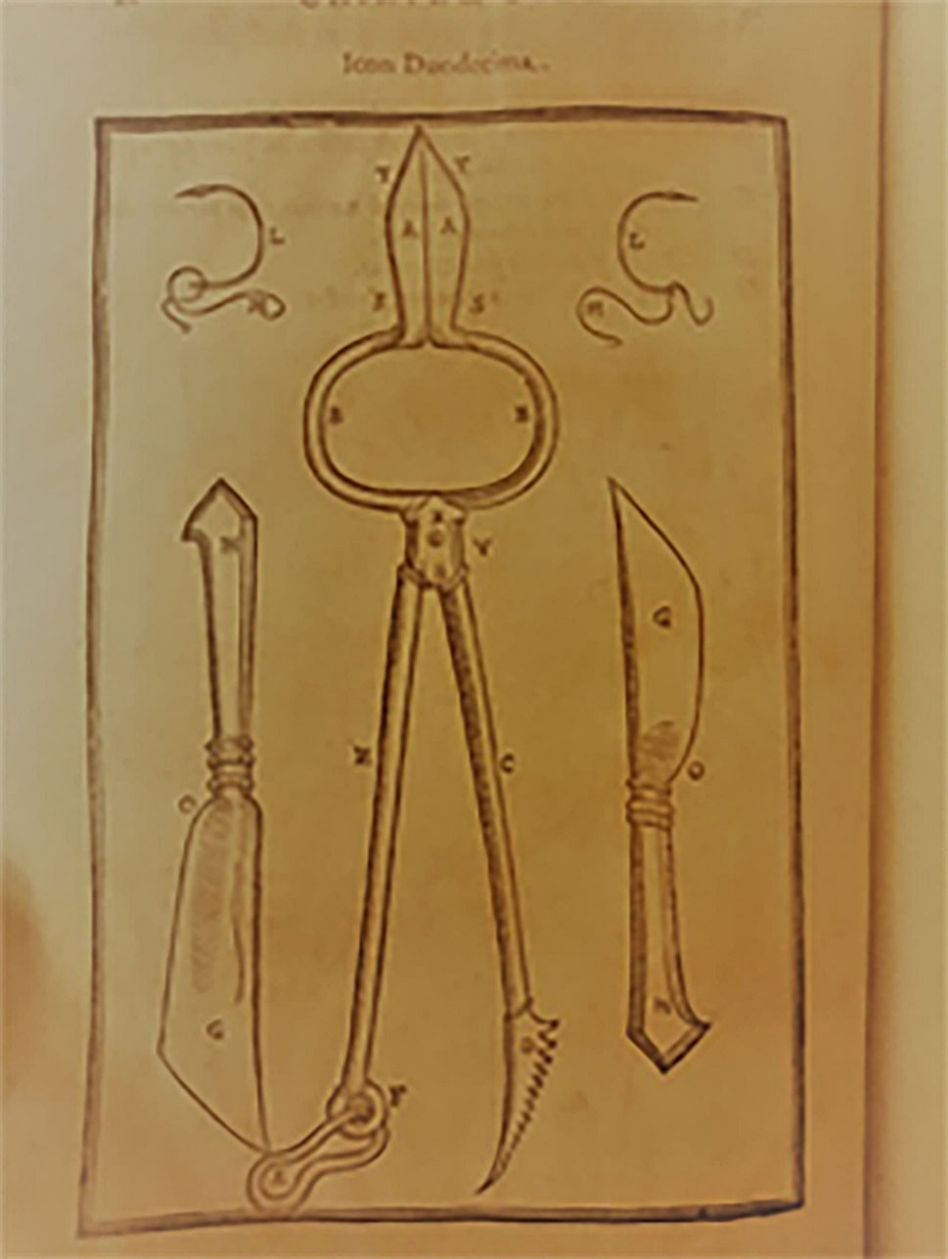
Surgical instruments, a special scissor with double use: holding the arm during the procedure while also cutting during flap harvest. Table 2, *De Curtorum chirurgia per insitionem, additis instrumentorum omnium et delegationum iconibus, et tebulis libri duo”.* Venezia, Bindoni, 1597, Tables 1, 2 and 12 (Library of History of Medicine—Sapienza University of Rome)

**Fig. 6:**
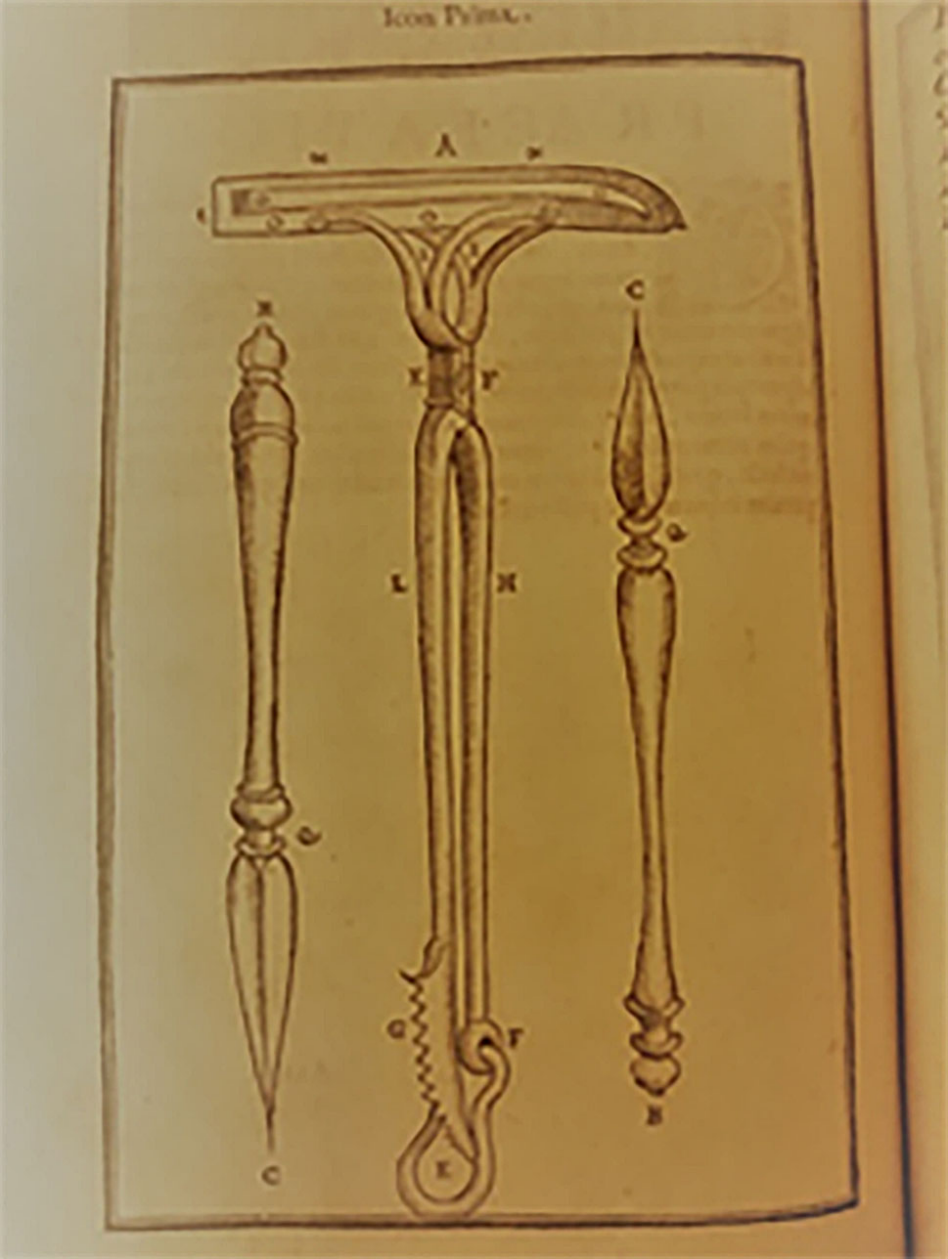
Surgical instruments, Table 12, *De Curtorum chirurgia per insitionem, additis instrumentorum omnium et delegationum iconibus, et tebulis libri duo”.* Venezia, Bindoni, 1597, Tables 1, 2 and 12 (Library of History of Medicine—Sapienza University of Rome)

**Fig. 7:**
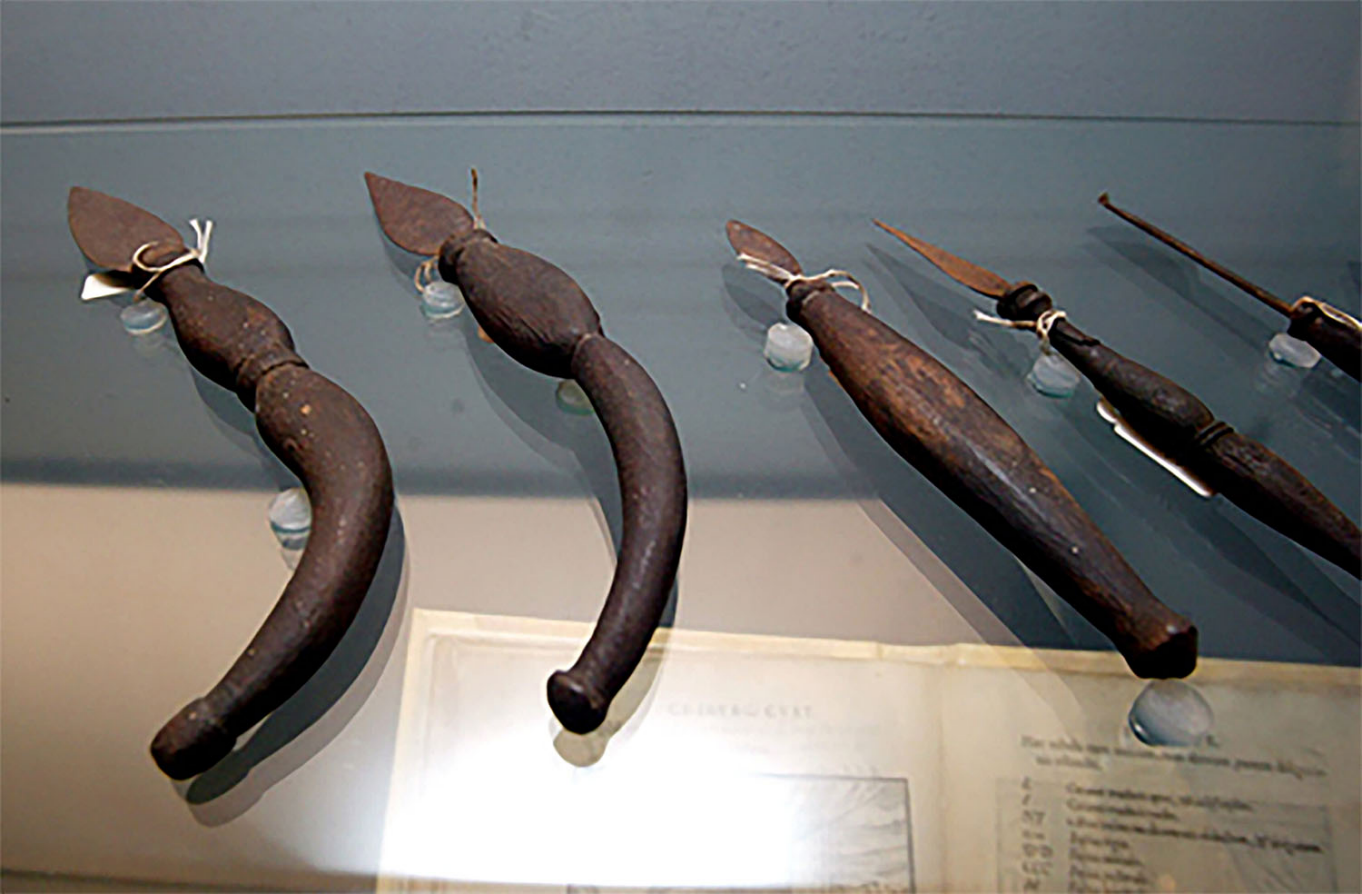
Surgical Instruments created by G. Tagliacozzi, surgical knives and a cautery. (Museum of History of Medicine—Sapienza University of Rome)

**Fig. 8:**
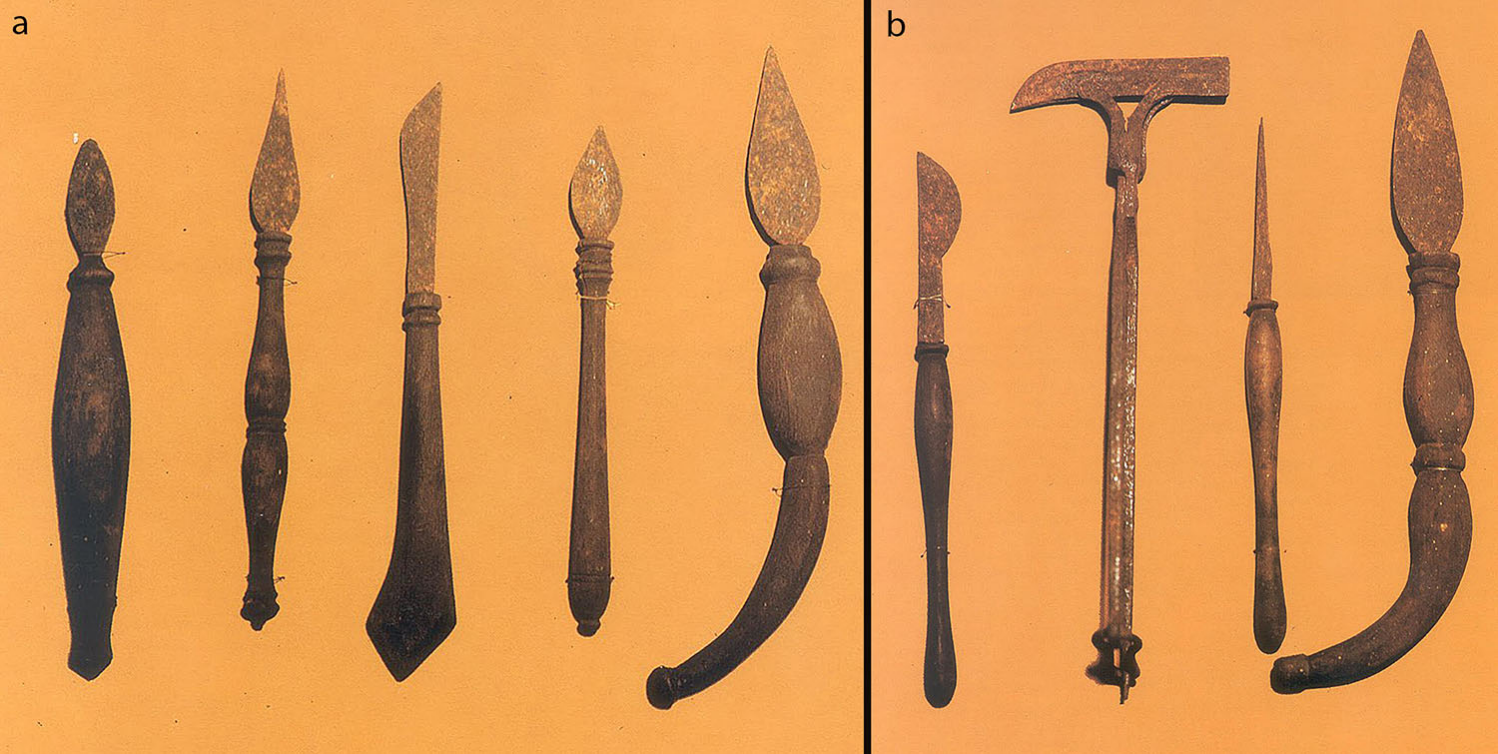
Surgical Instruments created by G. Tagliacozzi. **a** scalpels with leaf shape or half leaf shape and crescent shape. **b** A scalpel, a leaf shaped surgical knife, a surgical hammer also called “maglio” (double sided with a cutting edge on one side and a normal hammer on the other), and a pointed cautery (Museum of History of Medicine—Sapienza University of Rome)

He created for this procedure a set of instruments specifically designed to allow the skin flap from the arm to remain tightly placed over the nose (Figs. 4, 5, 6, 7, 8).

**Fig. 9:**
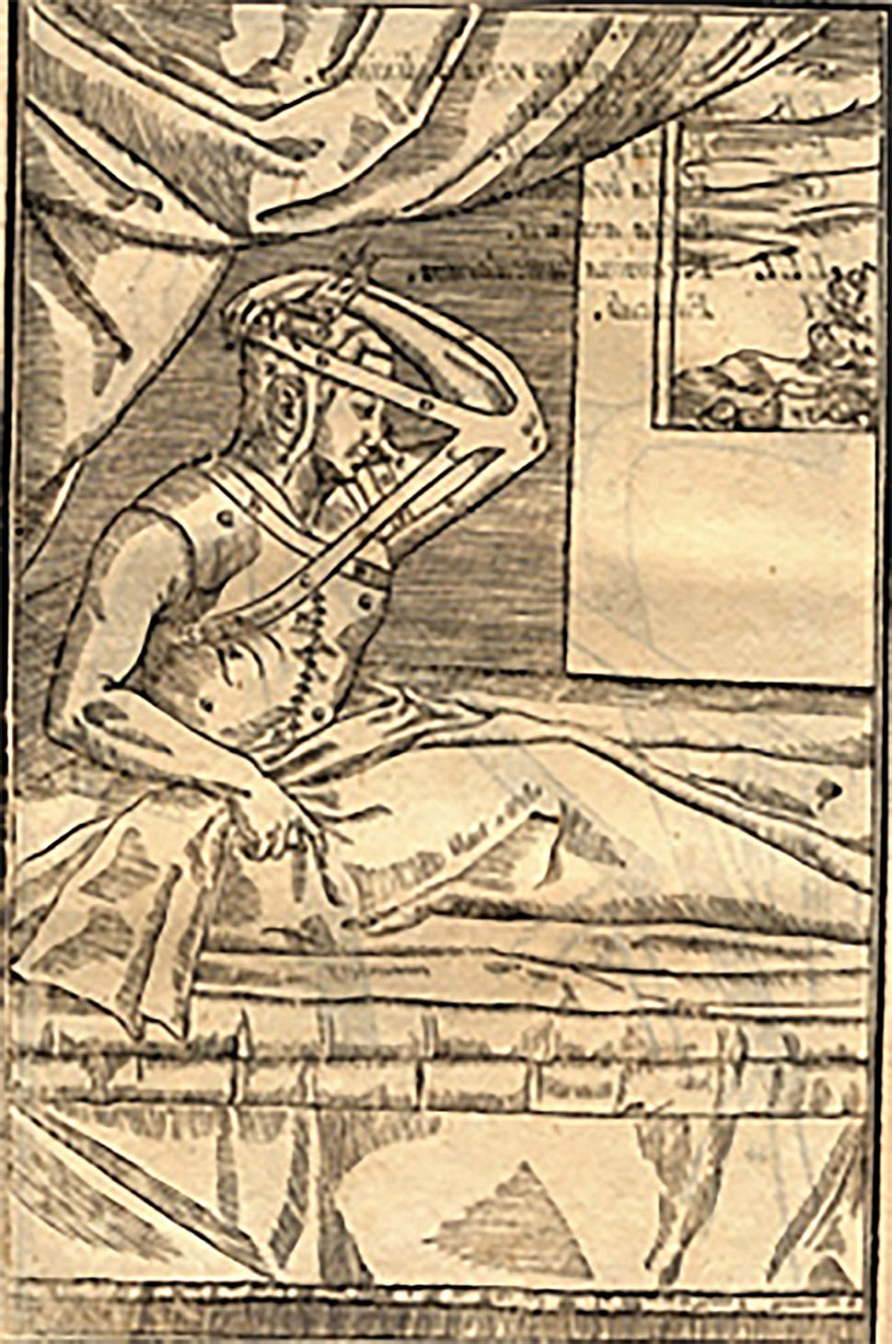
Tab. VIII from *De Curtorum chirurgia per insitionem, additis instrumentorum omnium et delegationum iconibus, et tebulis libri duo”.* Venezia, Bindoni, 1597. (Library of History of Medicine—Sapienza University of Rome)

**Fig. 10:**
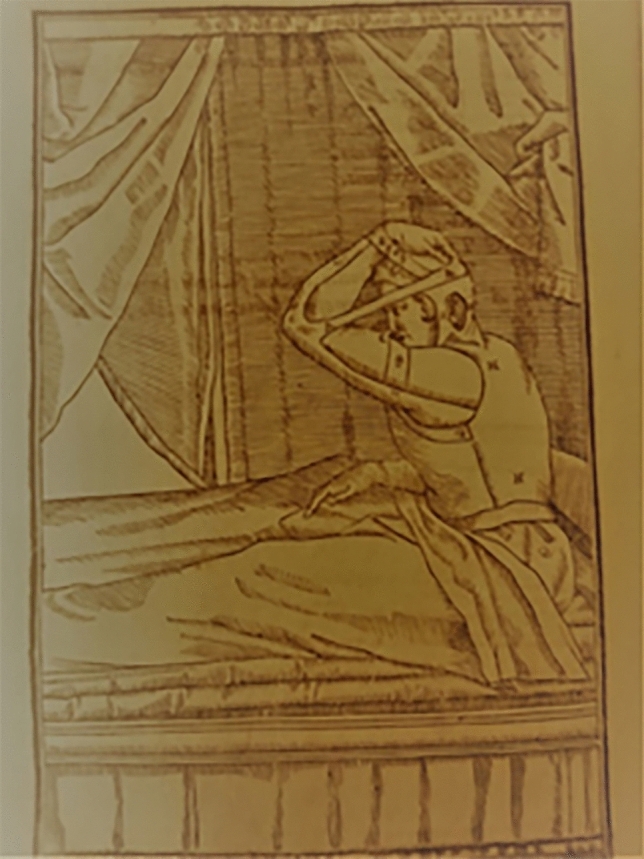
Tab. IX from *De Curtorum chirurgia per insitionem, additis instrumentorum omnium et delegationum iconibus, et tebulis libri duo”.* Venezia, Bindoni, 1597. (Library of History of—Sapienza University of Rome)

**Fig. 11:**
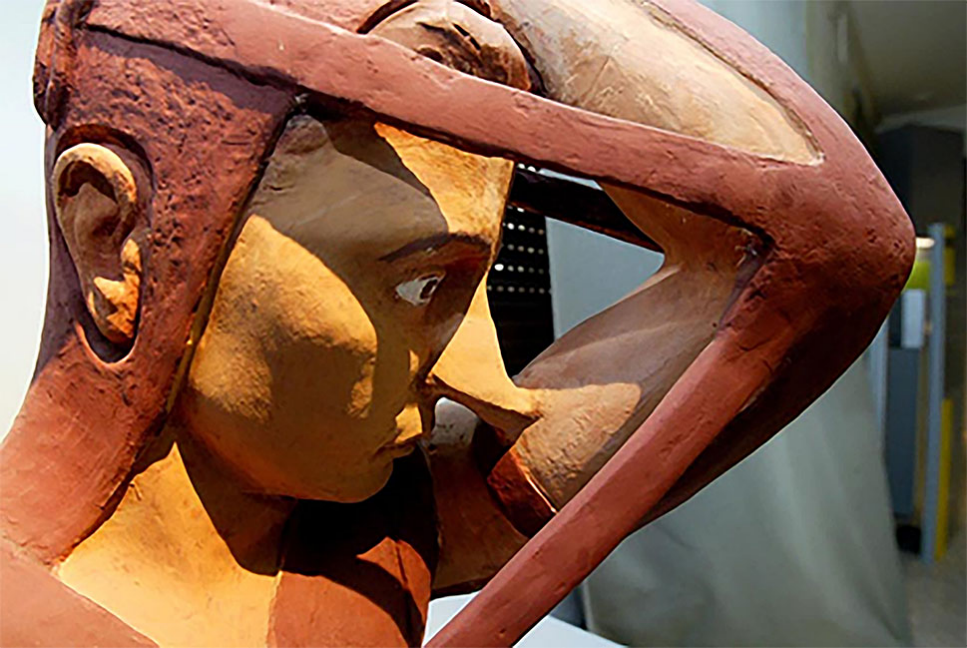
Bandage adopted by G. Tagliacozzi (Museum of History of Medicine—Sapienza University of Rome, Rome. Italy)

Among these, he mainly created and adopted a specific system of bandages, leather straps and metal structures well described over the past centuries. In fact, rhinoplasty as surgical technique was born as an evolution and refinement of the huge *corpus* of practical methods for bandaging and splinting the nasal traumatic injuries (Figs. 9, 10, 11) handed down since the earliest origins of plastic surgery in India to its modern development in Europe, passing through Greek , Roman, Byzantine and Arab periods.

Interestingly, in *De Curtorum* he makes a reference to agriculture in order to explain the concept of grafting, because he thought that there was an analogy “mutatis mutandis” between plant grafting and surgical grafting of human tissues.

In his works he reports that:

*[…]* *questi interventi sulle parti mutilate hanno avuto la loro ispirazione dalla agricoltura ed hanno avuto i loro inizi dall’innesto e si studiano di imitare la propagazione artificiale delle piante.* *[...]*

*[…]* *these interventions on the mutilated parts have been inspired by agriculture and have had their beginnings in grafting and are studied to mimic the artificial propagation of plants* *[...]* [14].

In this sense, he considers the cutaneous flap from the arm as something different from the nose but also similar, noticing how, in different plants, the propagation is possible by grafting one plant on the other, as long as there is an unspecified compatibility.

Tagliacozzi is, however, to be credited with having conferred an academic dignity in the field of surgery to an art until then bound to empirical practice, giving it its own body of doctrines and techniques. The consequence of Tagliacozzi's extensive practice of rhinoplasty led to an improvement in the methods and instruments, as testified by his “De Curtorum Chirurgia”, which systematized techniques, instruments, and therapies for rhinoplasty, thus founding a true specialistic surgical discipline [15, 16].

Rhinoplasty became officially the first field of autotransplantation, leaving to Tagliacozzi the credit, and the fault, of inspiring the idea of allotransplantation in an era not yet ready to accept it, despite the topic is still controversial nowadays.

The comparison that Tagliacozzi offers between the transplantation of a cutaneous flaps, harvested from a part of the body and insetted on another part, and plants grafting seemed to allow to theorize the possibility harvesting of cutaneous flaps also from other individuals. According to some historians, the practice of reconstructing the nose using autografting and/or skin flaps from other individuals had already become established and popular in the first half of the 16th century in various areas of Italy.

However, what is most relevant in Tagliacozzi’s work is not just the technical innovation, but the intuition that each human body has its own “peculiarities”, which make it difficult to merge with others.

The fame of the author of the *De Curtorum Chirurgia* opened the way to a medical and philosophical debate which, instead of bringing to adoption and advancements of the technique he proposed, would ultimately lead to prohibit it.

After his death, the rhinoplasty surgery performed by Tagliacozzi, which was difficult to apply, was abandoned for over two centuries. Many doctors and surgeons considered his surgery too painful, with results that were not always aesthetically satisfactory and, above all, lacking in scientific knowledge. However, the philosophical question that emerged around the exchange and connection between the soul and body of the donor and the receiver was a central topic of debate in the following years. In 1742, in fact, the Faculty of Paris prohibited its execution.

It is only in 1816 that Giuseppe Costantino Carpue [17, 18] resumed to perform surgery according to Tagliacozzi's model, opening the way to the affirmation of rhinoplasty as a real surgery, based on scientific bases and on expert knowledge. This, by the end of the Nineteenth century, allowed it to become a specialty within the broader category of Aesthetic and Reconstructive Plastic Surgery.

Therefore, the surgical technique of rhinoplasty developed firstly in Italy and, starting from the second half of the Nineteenth century, some interest started to grow in Northern Europe. Here, in 1845, Dieffenbach was the first to describe an attempt to reduce the size of a prominent nose by means of external incisions and soft tissue resection.

The interest shifted in 1887 in New York with Roe, who described a surgery performed from the inside of the nose limited to the remodeling of the tip, and in 1891  with the osteo-cartilaginous hump resection.

The intranasal approach described by Roe was then used also in 1898 by the famous German surgeon Joseph, considered the father of modern corrective rhinoplasty [19].

These exponents of the modern era of rhinoplasty are the heirs of a complex past in which East and West have strongly permeated each other as forerunners of the very modern concept of supranational medicine and culture, today essential and very stimulant for its great potential of confrontation and reciprocal enhancement: Science [20].

## References

[1] Avicenna (1608) Avicennae Arabum Medicorum Principis. Ex Gerardo Cremonense et Alpagi Bellunensi. Venetiis, apud Iuntas, Tom. II, Tract. III, chap. 19

[2] Bhishagratna KKL (1963) Sushruta Samhita English translation by, Calcutta, 1907. The Sushruta Samhita. [English translation based on the original Sanskrit text edited and published by Kaviraj Kunja Lal Bhishagratna.] . Varanasi, Chowkhamba Sanskrit Series Office, Chap. 16, 152–154

[3] La Rosa R, Tedesco A (1999) L’arte della Rinoplastica nell’Italia del Rinascimento. In: La Rosa R, Fibbi A, Staffieri A (Eds) Chirurgia funzionale ed estetica del naso. Planning Congressi, Bologna

[4] Nicaise E (ed) (1893) Chirurgie de Maitre Henri de Mondeville. Paris; Dottrina I, cap.1

[5] Micali G (1993). The Italian contribution to plastic surgery. Ann Plast Surg.

[6] Rolando dei Capezzuti (1498) La Chirurgia [i.e. Rolandina], Venezia

[7] Ms. Annali del mondo, vol. I, Biblioteca Domenicana di Palermo

[8] Greco M, Ciriaco AG, Vonella M, Vitagliano T (2010). The primacy of the Vianeo family in the invention of nasal reconstruction technique. Ann Plast Surg.

[9] Benedetti A (1502) Historia Corporis Humani sive Anatomice, Venezia, IV, 39

[10] Fioravanti L (1570) Il Tesoro della vita humana Diviso in libri quattro. Venezia, Gli Heredi di Melchior Sessa, L.II, c.27

[11] Fioravanti L (1570) Il Tesoro della vita humana. Diviso in libri quattro. Venezia, Gli Heredi di Melchior Sessa, L.II. cap.45

[12] Tagliacozzi G (1597) De curtorum chirurgia per insiemetionem. Venetiis, Gaspare Bindoni

[13] La Rosa R, Tedesco A (1999) L’arte della Rinoplastica nell’Italia del Rinascimento. In: La Rosa R, Fibbi A, Staffieri A (eds) Chirurgia funzionale ed estetica del naso. Planning Congressi, Bologna, nota 1, cap.I, p. 33

[14] Tagliacozzi G (1597) De Curtorum chirurgia per insitionem, additis instrumentorum omnium et delegationum iconibus, et tebulis libri duo”. Venezia, Bindoni, L.I, 12

[15] Tomba P, Viganò A, Ruggieri P, Gasbarrini A (2014). Gaspare Tagliacozzi, pioneer of plastic surgery and the spread of his technique throughout Europe in De Curtorum Chirurgia per Insitionem. Eur Rev Med Pharmacol Sci.

[16] Fortuna S (2002) Gaspare Tagliacozzi (1545–1599) e il trapianto nella chirurgia ricostruttiva del volto. In Interpretazione e individualità. Atti del XXI Colloquio sulla Interpretazione (Macerata, 26–27 marzo 2001), a cura di G. Galli. Macerata, Istituti editoriali e poligrafici internazionali, pp. 121–131

[17] Marinozzi S (1999) Vianeo e Gaspare Tagliacozzi; lo sviluppo della Rinoplastica nel XVI secolo [The Vianeo and Gaspare Tagliacozzi. The development of rhinoplasty in the XVIth century]. Med Secoli 11(3): 603–610 11624564

[18] Carpue CJ (1816) “An Account of Two Successful Operations for Restoring a Lost Nose (Lost Noses) from the Integuments of the Forehead, in the Cases of Two Officers in His Majesty's Army : To Which Are Affixed, Historical and Physiological Remarks on the Nasal Operation; Including Descriptions of the Indian and Italian Methods.” The Medico-Chirurgical Journal and Review vol. 4,23 (1817): 376–387

[19] Mazzola RF (1987). History of nasal reconstruction. A brief survey. Handchir Mikrochir Plast Chir.

[20] R Fibbi A (1999) Staffieri A (eds) Chirurgia funzionale ed estetica del naso. Planning Congressi, Bologna

